# Activation of Stellate Cells Before Induction of Hepatic Fibrosis – Precise Timing in Choline-deficient Diet-fed Rat Model

**DOI:** 10.1186/1476-5926-2-S1-S14

**Published:** 2004-01-14

**Authors:** Kazuhiko Besshi, Mutsunori Fujiwara, Haruki Senoo, Yasufumi Kondou, Yoshitaka Ohsugi, Taiji Hayashi, Kozo Ishidate

**Affiliations:** 1 Drug Safety Research Laboratory, Taiho Pharmaceutical Co, Ltd, 224-2 Ebisuno, Hiraishi, Kawauchi-cho, Tokushima, Japan; 2 Division of Pathology, Japanese Red Cross Medical Center, Japan; 3 Department of Anatomy, Akita University School of Medicine, Japan; 4 Department of Molecular Cell Biology, Medical Research Institute, Tokyo Medical and Dental University, Japan

## Introduction

Quiescent hepatic stellate cells (HSCs) store vitamin A as lipid droplets in the cytoplasm. The activated HSCs by several stimuli have functions similar to that of myofibroblasts and play key roles in hepatic fibrosis [1-3]. However, precise timing between activation of the HSC and induction of hepatic fibrosis is still unknown. Choline-deficient (CD) diet induces fatty liver and subsequently hepatic fibrosis in rats. We investigated the changes of HSCs in the progress of hepatic fibrosis induced by CD diet in rats, and analyzed the time course from the activation of the HSCs to the induction of hepatic fibrosis.

## Methods

Male Sprague-Dawley rats (Charles River Japan Inc.) were divided into two groups fed CD diet (DYET#118753; Dyets Inc.) for 8 weeks or standard laboratory diet (CE-2; CLEA Japan Inc.) as control. Food and water were available *ad libitum *throughout the study period. The rats were 6 weeks of age and weighing 150 to 170 g at start of CD diet administration. Four rats of each group were sacrificed under anesthesia at 2, 4, 6 and 8 weeks, respectively, after the beginning of CD diet administration. In addition, 4 rats as pretreatment-animal were sacrificed before CD diet administration.

Liver tissues, obtained from sacrificed rats, were fixed 2.5% glutaraldehyde and 1% osmium tetroxide and embedded in epoxy resin for electron microscopy. We counted microscopically the number of HSCs containing vitamin A lipid droplets in methylene blue-stained thick sections from epoxy-embedded tissue blocks. HSCs containing more than 10 vitamin A lipid droplets were defined as vitamin A-rich HSCs. Tissues for light microscopy were fixed in buffered 10% formalin and embedded in paraffin, and sections were stained with hematoxylin-eosin or silver impregnation. We observed development of fibrosis using these specimens. For detection of activated HSCs, sections for light microscopy were immunohisto-chemically stained with anti alpha-smooth muscle actin antibody (alpha-SMA monoclonal antibody, Dako Japan, Inc.). We counted microscopically the number of alpha-SMA positive HSCs.

The number of HSCs (/mm^2^) were shown as the mean – standard deviation. The data in CD diet group were compared with that in control group by t-test.

## Results

**Figure 1:**
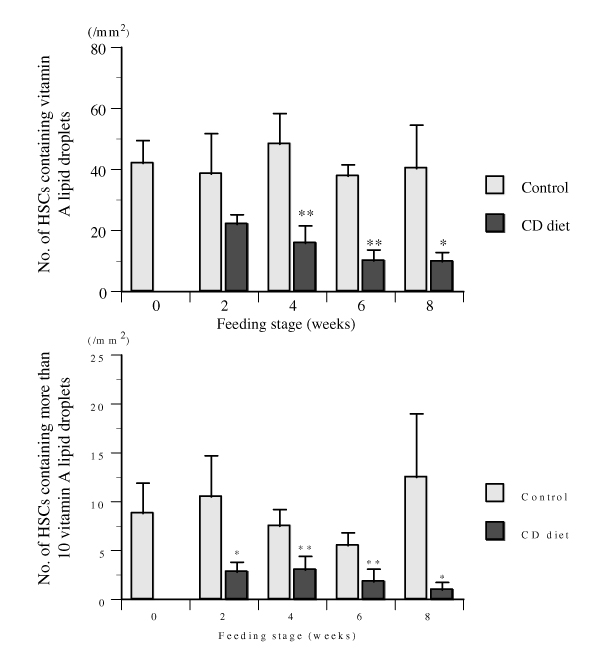
Number of hepatic stellate cells (HSCs) containing vitamin A lipid droplets in male rats fed choline-deficient (CD) diet for 2, 4, 6, and 8 weeks. Significant difference from control group (fed standard laboratory diet): *) p &lt; 0.05, **) p &lt; 0.01

The number of HSCs (/mm^2^) containing vitamin A lipid droplets are shown in figure 1. The number of HSCs containing vitamin A lipid droplets in CD diet group after 2 weeks-feeding was fewer than that in control group, however, there was no statistically significant difference between two groups. Thereafter, the number of HSCs in CD diet decreased further, and were fewer with significant difference (p &lt; 0.01 or 0.05) at 4, 6 and 8 weeks after the beginning of CD diet administration. The number of vitamin A-rich HSCs, containing more than 10 vitamin A lipid droplets, in CD diet were fewer with significant difference (p &lt; 0.01 or 0.05) after 2, 4, 6, and 8 weeks-feeding.

**Figure 2:**
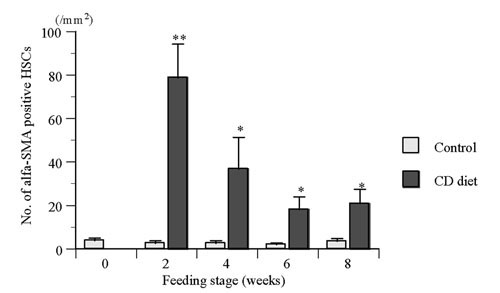
Number of alpha-smooth muscle actin (alpha-SMA) positive hepatic stellate cells (HSCs) in male rats fed choline-deficient (CD) diet for 2, 4, 6, and 8 weeks. Significant difference from control group (fed standard laboratory diet): *) p &lt; 0.05, **) p &lt; 0.01

The number of alpha-SMA positive HSCs (/mm^2^) are shown in figure 2. The number of alpha-SMA positive HSCs in CD diet group were larger with significant difference (p &lt; 0.01 or 0.05) than that in control group after 2, 4, 6, and 8 weeks-feeding.

In histopathological examination using specimens stained with hematoxylin-eosin or silver impregnation, CD diet group showed no fibrosis after 2 weeks-feeding, but showed slight fibrosis in the pericentral and periportal regions after 4 weeks-feeding. Fibrosis in CD diet group was progressive, and pseudolobules were formed after 8 weeks-feeding. In addition, CD diet group showed cytoplasmic vacuolization of hepatocytes (fatty change) from 2 weeks to 8 weeks-feeding.

## Discussion

Quiescent HSCs store vitamin A as lipid droplets in the cytoplasm. The HSCs are transformed to myofibroblast-like cells when showing a decrease in number of vitamin A lipid droplets, activated by several stimuli and express alpha-SMA [1-5].

In our present study, the number of HSCs containing vitamin A lipid droplets decreased, and alpha-SMA positive HSCs increased after 2 weeks-feeding CD diet. However, CD diet group showed histologically no hepatic fibrosis after 2 weeks-feeding. Thereafter, slight fibrosis in the pericentral and periportal regions was observed after 4 weeks-feeding. Fibrosis was progressive, and pseudolobules were formed after 8 weeks-feeding CD diet.

It is reported that hepatic vitamin A decreases in hepatic fibrosis and cirrhosis [5,6]. In rat and human, approximately 90% of total body vitamin A is stored in the liver [7]. HSCs take up retinol-retinol binding protein as vitamin A from blood stream [8], and store approximately 75% of the total hepatic vitamin A [7]. It was unclear whether decrease of vitamin A in HSCs in fibrosis/cirrhosis precedes the induction of fibrosis/cirrhosis or is the consequence of fibrosis/cirrhosis. Our study is the first report demonstrating that transformation of HSCs from quiescent to activated phenotype precedes 2 weeks before induction of fibrosis in the CD diet-fed rat model, and suggests that this transformation of HSCs is a cause of hepatic fibrosis.
